# The High Throughput Sequence Annotation Service (HT-SAS) – the shortcut from sequence to true Medline words

**DOI:** 10.1186/1471-2105-10-148

**Published:** 2009-05-16

**Authors:** Szymon Kaczanowski, Pawel Siedlecki, Piotr Zielenkiewicz

**Affiliations:** 1 Bioinformatics Department, Institute of Biochemistry and Biophysics, Polish Academy of Sciences, ul Pawinskiego 5a, 02-106 Warszawa, Poland; 2 Department of Plant Molecular Biology, Institute of Experimental Plant Biology, University of Warsaw, Miecznikowa 1, 02-096 Warsaw, Poland

## Abstract

**Background:**

Advances in high-throughput technologies available to modern biology have created an increasing flood of experimentally determined facts. Ordering, managing and describing these raw results is the first step which allows facts to become knowledge. Currently there are limited ways to automatically annotate such data, especially utilizing information deposited in published literature.

**Results:**

To aid researchers in describing results from high-throughput experiments we developed HT-SAS, a web service for automatic annotation of proteins using general English words. For each protein a poll of Medline abstracts connected to homologous proteins is gathered using the UniProt-Medline link. Overrepresented words are detected using binomial statistics approximation. We tested our automatic approach with a protein test set from SGD to determine the accuracy and usefulness of our approach. We also applied the automatic annotation service to improve annotations of proteins from *Plasmodium bergei *expressed exclusively during the blood stage.

**Conclusion:**

Using HT-SAS we created new, or enriched already established annotations for over 20% of proteins from *Plasmodium bergei *expressed in the blood stage, deposited in PlasmoDB. Our tests show this approach to information extraction provides highly specific keywords, often also when the number of abstracts is limited. Our service should be useful for manual curators, as a complement to manually curated information sources and for researchers working with protein datasets, especially from poorly characterized organisms.

## Background

High throughput experiments such as sequencing projects, microarrays and proteomic methods produce huge amounts of data, usually in the form of long lists of genes or proteins. Proper annotation of such lists is crucial for understanding and interpretation of experimental results.

The best annotation quality is still generated by curators who manually review the existing literature. This approach for describing genes and/or their products is utilized by most large projects like FlyBase [1], UniProt [2] and others. The best known and most widely used public annotation database is Gene Ontology (GO) [3], which maintains a controlled vocabulary (called terms) organized into three hierarchies: biological process, molecular function and cellular component. These GO terms are utilized by e.g. the GOA project [4] that provides Gene Ontology annotations for the UniProt database, International Protein Index (IPI) and other major databases such as Ensembl and NCBI. All GO annotations can also be searched by gene/protein name or GO term using the AmiGO [5] official browser.

Beside Gene Ontology annotations, manual descriptions are also generated by a number of other projects. Examples include KEGG [6] which is focused on metabolic pathways, and UniProt which provides, in the comments section, biological knowledge usually directly linked to publications.

The existing manual annotation systems, despite their undoubted importance, are also acknowledged to have serious limitations. One of the main obstacles of using manually curated descriptions is that high coverage is available mostly in very broad categories. In the case of GO, diving deeper into their DAG structure shows the annotations are not of equal quality and a large proportion of them come from automatic computer predictions (i.e using motif databases or simple sequence similarity) [7]. Broad protein classes (methyltransferases, kinases, phosphatases, etc) are assigned and are automatically inherited if a "new" protein has a domain with a GO label attached. As manually curated annotation databases are notoriously incomplete (see e.g. [7]) this raises the problem of using more specific GO terms for statistical analysis of gene datasets.

Another hindrance is that many genomes are poorly, or not at all covered by GO terms (or other manually curated annotations), even for such prominent species as *Gallus gallus *or the slime mold [8]. Computational methods have been developed to at least partially overcome the coverage problems mentioned. BLAST2GO [9] or KAAS [10] both based on sequence similarity, help "transfer" annotations between orthologious genes/proteins. Another way to circumvent the coverage problem is to use published information about genes/proteins and automatically categorize them based on specific features (e.g. combinations of keywords/terms). Some services that analyze texts in this aspect include GOCAT [11] which can categorize any text according to its similarity to GO vocabulary, or GOAnnotator [12] which acts similarly by fetching possible text evidences for electronic GO annotations.

Such approaches generally give quite good results and are currently intensively being developed. Better performance is reached by introducing new aspects in the scoring methods, such as proximity between words in text and weighing words according to the amount of information they carry [13]. It is possible to compare various computational approaches in a contest organized by the BioCreAtive initiative [14].

Unfortunately using annotations based on GO terms has one more serious disadvantage, which is the paucity of the language used. Gene Ontology categorizes language in order to decrease its complexity and to provide a unified way to describe certain biological aspects. Although in some cases this controlled vocabulary brings significant advantages, it also *de facto *means that annotators have to discard specific information or features present in publications in order to comply with a GO term [3,7]. This becomes a highly visible problem when one tries to differentiate between proteins with similar but not identical functions – (e.g H4 and H2B-Alpha from *S. pombe*). Also not all of the concepts are covered by the GO thesaurus (e.g. cellular component-apicoplast).

Nevertheless, GO terms are frequently used to analyze sets of genes to discover over-representation of particular functions or categories in the dataset. Dedicated software such as GeneMerge [15] or GeneTools [16] is available for this type of analysis.

We still lack annotation services, which could annotate genes/proteins using sequence homology and multiple sources of information (created both manually and automatically). Popular services like SMART [17], PFAM [18] or PROSITE [19] can be searched using sequence homology but they gather only manual annotations (manually described protein domains and protein motifs). InterPro [20] from EBI combines most of the different protein signature databases into one sequence analysis service. With IntePro2go [21] one can additionally associate GO terms with different InterPro entries. The main problem with this system is the contrasting specificity of IntePro signatures: some of them are very frequent in nature and associated with very broad molecular functions, while others are highly specific. Also all these services/databases use manually curated information more or less shared among them.

Taking into account the decreasing costs of high-throughput experiments and the increasing number of organisms studied, there seems to be an expanding gap between available manual annotations and the demand to describe novel genomes, proteomes, etc. A promising way to bridge this gap is to develop and/or improve methods for automatic annotation generation. Such methods should be based both on available manual curation and on automatic information extraction from other sources. Also a recent publication by Jaeger *et. al. *[22] shows other types of data such as conserved networks (including ortholog) can be utilized to create functional annotations based on literature analysis.

Taking the literature into consideration brings advantages to the annotation process: publication abstracts are currently the vastest source of biological data available, which grows almost exponentially and contains information not yet found or described by manual curators. It is also valuable that various statistical approaches can be applied to extract informative and specific textual features from a set of documents [23,24]. Additionally, by processing the "raw" texts we omit the language paucity problem and enrich the retrieved knowledge with additional keywords like frequently used synonyms of gene names, chemical compounds, functionally interacting proteins, etc.

There are services implementing this idea. MedBlast [25] is a simple web-based application which can gather Medline abstracts connected with a given gene. It uses BLAST to find abstracts linked to homologous sequences but can also find abstracts with the gene and organism name derived from annotated protein entries. The output is a set of Medline documents which should be read by the user. A more sophisticated approach was proposed by Attwood and co-workers. METIS [26] also uses BLAST homology search to gather literature (abstracts), but the collected text is passed into two sentence classification components (set of SVMs and BioIE [27]). They find and categorize informative sentences, relating them to: structure, function, disease and localization categories. METIS also calculates simple statistical properties, i.e. n-grams of word distribution and frequently occurring words. Unfortunately METIS is unable to annotate more than one protein during one run. It also displays only frequently occurring words, which often appear in the whole Medline, thus are neither very informative nor specific, e.g. cell, protein, etc.

To help automate the annotation of a large set of proteins using literature derived knowledge we created HT-SAS (High Throughput Sequence Annotation Service). It brings homology search and textual feature extraction from literature together and is easily operated through a user friendly web interface. The input for HT-SAS are protein sequences. Homology search is performed using BLAST algorithm [28] against the UniProt database. HT-SAS utilizes links between UniProt protein entries and Medline to gather publication abstracts. The obtained sets of abstracts are searched for statistically important words using a modified TF-IDF approach. This feature extraction approach is more informative than simple word frequency and provides specific keywords aiding the annotation task. It can often provide significant keywords even for a limited number of abstracts, which is usually the case for poorly described or unknown proteins.

The interactive web interface allows easy access to source publications, homology results, and also Gene Ontology and UniProt manual annotations, if available. The results page is generated in the form of sortable tables for fast result overview and comparison. The URL is: 

## Implementation

### Aim of the system

The aim of the algorithm is to find biologically significant information (i.e. which can be used to annotate unknown proteins) in freely available literature by combining a sequence similarity search with a statistical analysis of textual features. This idea is based on the assumption that words appearing frequently in abstracts linked with a set of proteins, and which rarely appear in the whole abstract database, are specific to these proteins and thus can have biological meaning. Our algorithm uses a probabilistic approach to evaluate statistical significance of word appearance.

### Statistical approach

Every protein (p) has its own document frequency (df_p_), i.e. the number of abstracts which are linked to this protein. Sequence similarity is used to define sets of proteins related to the user provided protein sequence (query). HT-SAS identifies protein homologs using the BLAST algorithm. The sensitivity of the search, i.e. the cutoff of accepted homology, is defined by the user with the BLAST e-value parameter.

Using BLAST we obtain a set of homologous proteins (sp) and count the number of documents that relate to this set of proteins (df_sp_). Next we calculate the document frequency of all documents linked to a set of proteins and containing a given word (df_wsp_).

To properly estimate the background probability of a given word (p_w_) we created a subset of Medline (called MUDB), which stores only abstracts that are linked to UniProt proteins. This ensures that we avoid word bias by including documents not referring to proteins. In our approach the background probability (p_w_) is the number of documents in MUDB containing a given word (document frequency of a word – df_w_), divided by the total number of documents deposited in MUDB. This is in fact similar to a classical IDF-like approach [29] which discriminates words occurring frequently (e.g. cancer, protein, cell, etc.).

Now we can finally calculate the probability of word occurrence (P_wo_) using the following formula:

(1)

By using such an approach we are able to extract interesting, specific textual features, but there is also a significant amount of "noise" present. By "noise" we mean very broad and not very useful words such as yeast, two-hybrid, etc. We found this effect to be caused by documents pointing to a huge number of proteins, e.g. publications describing sequencing projects or other high-throughput experiments – in the majority of cases such abstracts do not contain specific information. To resolve this problem abstract weight was introduced. It is calculated by dividing one by the number of proteins an abstract points to. Therefore df_sp _and df_wsp _become in fact the sum of document weights (wdf_sp _and wdf_sp _respectively) and are rounded to an integer. This normalization parameter ensures that documents linked to many proteins (outliers) are discriminated and have lower impact on the calculation.

(2)

The last modification is based on keyword frequency distribution observed in documents deposited in MUDB. The number of analyzed abstracts linked to a set of homologous proteins in the majority of cases is not greater than 10^2^. This is significantly smaller than the total number of abstracts deposited in MUDB which is in the order of 10^5^. We assume that biologically significant and specific words occur in abstracts not more frequently than 10^-2- ^10^-4 ^(e.g. "histone" occurs in about 2500 abstracts out of more than 200 000 abstracts which are linked with UniProt). This implies a small background probability which allows the following approximation shown below:

(3)

Without the need of extensive calculations (which do not significantly influence the annotation words statistics – *see * for details) we are able to annotate faster and provide a more pleasant user experience with a snappier interface.

### Database details

We created a subset of Medline abstracts linked to UniProt entries (i.e. PMID accession numbers derived from the id field of <dbReference type="PubMed"/> from UniProt XML files) called MUDB. MySQL database has been used to store various information regarding this subset. The most important are the number of proteins linked with a publication, word frequency (words are taken from <title> and <abstract> fields of Medline XML files, multiple usage of a word in the title and abstract is counted as one to reduce the noise level) and information derived from UniProt <protein>, <comment> and <dbReference type="Go"> fields.

To keep MUDB up-to-date, Medline and UniProt files are updated twice a month.

Obviously publications linked to UniProt protein entries are only a fraction of the full list of literature concerning a particular protein. Nevertheless they are carefully chosen manually to provide comprehensive and non-redundant knowledge. It is also important that HT-SAS uses all abstracts linked with the homolog list, so overall the gathered literature should cover the majority of knowledge about a protein set, and thus be sufficient to annotate a new sequence.

### Web interface

The service is designed for a wide range of users, including those who are not involved in protein research. The user is asked to provide protein sequence(s) in fasta format. The system automatically checks if user data is properly formatted and if it is likely to be a protein. If errors are encountered at this stage, HT-SAS allows the user to correct the data and resubmit. Otherwise a temporary file is saved and the user can adjust the BLAST e-value parameter. The e-value parameter is essential in this literature mining approach. It defines the level at which proteins are considered similar and therefore directly influences the number of abstracts to be analyzed. Theoretically if high values are chosen (> 1e-20) then the system recognizes even very distantly related proteins as homologous, and as such, should analyze a larger set of abstracts. The downside is it will report information that might be somewhat "noisy". If smaller e-values are chosen, HT-SAS will find fewer homologs, resulting in a smaller set of analyzed abstracts.

After the BLAST e-value is chosen the user can start the literature mining engine and monitor the annotation progress through the web interface. When all of the sequences are annotated a results page is displayed which shows separate tables for each protein sequence submitted. Each table has the sequence name in its title and a list of words associated with it ("annotation keywords"). Each keyword is associated with its "Score" which describes how specific and significant it is (calculated with formula 2). Through these tables the user can access more specific data such as lists of (linked) publications where the word was present, lists of homologous proteins (UniProt IDs) with alignments, lists of UniProt words and Gene Ontology descriptions if present.

If the user submitted multiple sequences, HT-SAS allows comparing annotations. The web interface will show which words are shared between which sequences, helping the researcher to quickly group them based on the occurring terms.

## Results

### Evaluation by precision and recall

We have conducted test experiments to assess the quality and usefulness of keywords obtained by HT-SAS. In the first experiment we checked if our algorithm can sufficiently recreate knowledge available in a manually curated database. We used proteins deposited in the Saccharomyces Genome Database (SGD, [30]) as the majority of *S. cerevisiae *genes are well described and could be used as reference annotations. We have randomly drawn 100 sequences from SGD which were next automatically annotated using HT-SAS with a default "mining threshold" value [20].

From this set 63 sequences had been annotated by HT-SAS with at least 5 keywords which had "Score" ≤ -10 (*P*-value equal or smaller than 10^-10^). This keyword scheme was sufficient to manually recreate the original annotations [see Additional file 1 for details], indicating a recall of 63%. We also measured the precision of obtained annotations. One of 5 keywords from each sequence was randomly chosen and manually evaluated. In 54 cases these keywords were correct and accurate, giving an overall precision of over 85% [see Additional file 1 for details].

We also asked the question what would happen to our automatic keyword extraction if these random proteins did not have any close homologs. We conducted the same experiment, filtering out homologs with more than 50% sequence identity. In this case recall (measured as above) dropped to 42% but precision stayed high (40/42, over 92%) [see Additional file 2 for details].

In case of 4 proteins HT-SAS provided keywords suggesting interesting hypotheses that:

a) uncharacterized protein YNR065C is a hydroxysteroid dehydrogenase

b) protein of unknown function YJR134C is a myosine like protein

c) kinase YMR291W is a calmodulin dependent kinase

d) YNR065C is related to sortilin receptor like protein

These results show that HT-SAS correctly selects words which are important and specific for protein description (recall of 42–63%) and that the keyword extraction algorithm works properly, even if the number of homologous proteins is limited (precision of 85–92%).

### Case study: Annotation of genes expressed exclusively during blood stages of *Plasmodium bergei*

To further assess the usefulness of HT-SAS service we conducted a test analysis of a protein set from a poorly described organism. This would mimic one of the major applications of HT-SAS – a service designed to enhance information associated with protein sequences deposited in databases. If the user has a set of interesting proteins and some of them are poorly characterized, they can be searched with HT-SAS for additional/novel keywords derived directly from literature.

In this case study we have used a set of 171 genes identified exclusively during blood stages of *P. berghei *[31]. Plasmodium proteins are generally poorly annotated compared to other model organisms, although there is much ongoing research and published literature. Manual annotations associated with these proteins were taken from two sources: PlasmoDB [32], which is the primary source of information about P. *bergei *genes and UniProt.

Using HT-SAS we obtained annotation keywords for 98 genes (keywords which had "Score" ≤ -5). In this set we were able to improve the PlasmoDB and UniProt annotations using novel keywords provided by HT-SAS for 11 proteins. Also using literature-derived keywords we were able to build new annotations for 24 proteins which are termed "hypothetical protein", "putative uncharacterized protein", etc. [see Additional file 3 for details]. This result shows that for over 20% of proteins in this set HT-SAS was able to provide information which was sufficient to augment existing annotations or create novel ones. This demonstrates the usefulness of our approach not only to manual curators but also to regular users which can test whether HT-SAS can augment annotations of their protein(s).

## Conclusion

Both sources of information – manually curated annotations and literature can complement each other and can both provide significant information helping to describe genes/proteins. The software presented here provides a way to quickly generate an overview of the relevant literature in a form of keywords which are directly linked to source abstracts. Our automatic keyword extraction approach gives good precision and recall and could be useful for those who are working with sparsely annotated genomes and large genome datasets.

## Availability and requirements

Project name: HT-SAS

Project home page: 

Operating system: platform independent

Programing language: PERL/CGI

License: GNU GPL (upon request)

This service is freely available. Due to the limit of our infrastructure one user can annotate 100 sequences per day. If there is a need for more computation power please contact the authors directly.

## Authors' contributions

Both SK and PS have equally contributed to the work. PS has designed the web interface. PZ has made substantial contributions to conception, design and interpretation of data. All authors read and approved the manuscript.

## Supplementary Material

Additional file 1 **Evaluation of HT-SAS using 100 random proteins from SGD**. Data provided represents the evaluation of HT-SAS annotations compared with manually curated descriptions from SGD. Click here for file

Additional file 2 **Evaluation of HT-SAS using 100 random proteins from SGD. Query homologues with sequence identity > 50% were excluded**. Data provided represents the evaluation of HT-SAS annotations when close homologues were excluded, compared with manually curated descriptions from SGD. Click here for file

Additional file 3 **Annotation of genes expressed exclusively during blood stages of *Plasmodium bergei *using HT-SAS**. Data provided compares PlasmoDB, UniProt and HT-SAS annotations of Plasmodium genes, expressed exclusively during blood stages. Click here for file
